# Refinement of ectopic protein expression through the GAL4/UAS system in *Bombyx mori*: application to behavioral and developmental studies

**DOI:** 10.1038/s41598-017-12102-2

**Published:** 2017-09-18

**Authors:** Chiho Hara, Koudai Morishita, Seika Takayanagi-Kiya, Akihisa Mikami, Keiro Uchino, Takeshi Sakurai, Ryohei Kanzaki, Hideki Sezutsu, Masafumi Iwami, Taketoshi Kiya

**Affiliations:** 1 Division of Life Sciences, Graduate School of Natural Science and Technology, Kanazawa University, Kakuma-machi, Kanazawa, Ishikawa 920-1192 Japan; 2 Research Center for Advanced Science and Technology, The University of Tokyo, 4-6-1 Komaba, Meguro-ku, Tokyo 153-8904 Japan; 3 Transgenic Silkworm Research Unit, Institute of Agrobiological Sciences, National Agriculture and Food Research Organization, 1-2 Owashi, Tsukuba, Ibaraki 305-8634 Japan

## Abstract

Silkmoth, *Bombyx mori*, is one of the important model insects in which transgenic techniques and the GAL4/UAS system are applicable. However, due to cytotoxicity and low transactivation activity of GAL4, effectiveness of the GAL4/UAS system and its application in *B. mori* are still limited. In the present study, we refined the previously reported UAS vector by exploiting transcriptional and translational enhancers, and achieved 200-fold enhancement of reporter GFP fluorescence in the GAL4/UAS system. Enhanced protein expression of membrane-targeted GFP and calcium indicator protein (GCaMP5G) drastically improved visualization of fine neurite structures and neural activity, respectively. Also, with the refined system, we generated a transgenic strain that expresses tetanus toxin light chain (TeTxLC), which blocks synaptic transmission, under the control of GAL4. Ectopic TeTxLC expression in the sex pheromone receptor neurons inhibited male courtship behavior, proving effectiveness of TeTxLC on loss-of-function analyses of neural circuits. In addition, suppression of prothoracicotropic hormone (PTTH) or insulin-like peptide (bombyxin) secretion impaired developmental timing and growth rate, respectively. Furthermore, we revealed that larval growth is sex-differentially regulated by these peptide hormones. The present study provides important technical underpinnings of transgenic approaches in silkmoths and insights into mechanisms of postembryonic development in insects.

## Introduction

Transgenic techniques that enable expression of endogenous or exogenous genes in desired cells or tissues are essential for current biological researches. In the vinegar fly, *Drosophila melanogaster*, introduction of the GAL4/UAS system that utilizes yeast transcription factor GAL4 and its binding sequence, upstream activating sequence (UAS), greatly expanded the utilities of transgenic approaches^1^. These approaches made precise manipulation of transgene expression possible even at single-cell resolution and became powerful tools^2^. In silkmoths, *Bombyx mori*, establishment of *piggyBac*-mediated transgenic techniques^3^ and the GAL4/UAS system^4^ also contributed to a wide range of scientific studies^5–8^. In addition, *B. mori* is a useful organism for various industrial studies. For example, taking advantage of its large body size and easiness of rearing, *B. mori* is used for mass production of eukaryotic proteins^9,10^. However, application of the GAL4/UAS system in *B. mori* was often hampered by the cytotoxicity and relatively low transactivation activity of GAL4. Previously, to overcome these technical obstacles, a series of GAL4 variants that have strong transactivation activity were tested for their applicability in silkmoths^11^. Although some GAL4 variants showed high transactivation activity in *B. mori* cells, these variants were not useful for generating transgenic silkmoth strains, because cytotoxicity correlatively increased with the transactivation activity. Thus, effective methods to generate transgenic silkmoths that have strong transgene expression through the GAL4/UAS system have not yet been established. Recently, comprehensive optimizations of targeted gene expression systems, including the GAL4/UAS system, were conducted in *D. melanogaster* ^12,13^. In these reports, not only GAL4 optimization but also addition of transcriptional and translational enhancers to UAS vectors significantly improved transgene expression. These results prompted us to examine the possibility that refinement of UAS vector improves transgene expression while avoiding GAL4 cytotoxicity in *B. mori*, and further explore its effectiveness in studies of sexual behavior and development.

Sex pheromone plays essential roles in proper and efficient mate recognition in many animals^14^. In *B. mori*, sexual behavior of males is regulated by sex pheromones emitted from females, which consist of major and minor components, bombykol [(E,Z)-10,12-hexadecadien-1-ol] and bombykal [(E,Z)-10,12-hexadecadien-1-al], respectively^15,16^. Sex pheromone communication of *B. mori* is simple where every component of courtship behavior is elicited by bombykol. Airborne bombykol is detected by a specific odorant receptor, BmOR1, expressed in the antennae^17^. BmOR1-expressing neurons project their axons to the antennal lobe, which is the primary olfactory center, and converge onto a specific glomerulus, toroid^18^. Bombykol information is further processed in the higher brain areas and finally elicits courtship behavior^18^. Since robust behavioral response is induced by a single substance, silkmoth brain serves as an ideal model for investigating neural circuits that regulate a behavior. In fact, extensive studies have been conducted to understand the neural mechanisms of how sex pheromone regulates courtship behavior in *B. mori* ^5,6,8,17–20^. However, though these studies revealed candidate neural circuits and mechanisms responsible for the behavior, *in vivo* analyses using transgenic silkmoths to prove sufficiency and necessity of the neural circuits are still lacking.

In insect development, final body size is determined by concerted actions of ecdysone and insulin signaling^21–25^. In holometabolous insects, pulsed increases of 20-hydroxyecdyone (20E), the active metabolite of ecdysone, induce molting and metamorphosis. Ecdysone is biosynthesized and secreted from the prothoracic glands (PGs), whose activity is regulated by prothoracicotropic hormone (PTTH)^26^. In contrast, insulin-like peptide, which is called bombyxin in *B. mori*, promotes blood sugar uptake and controls systemic growth^27–29^. Thus, PTTH and bombyxin play important roles in determining the timing of molting and metamorphosis (growth period) and the rate of gaining weight (growth rate), respectively. These two peptide hormones are produced in distinct neurosecretory cells in the brain and secreted from corpora allata to hemolymph in *B. mori* ^30,31^. Although both PTTH and bombyxin were originally purified from *B. mori* ^26,29^ decades ago, their importance *in vivo* has not been investigated. (However, while we were preparing this manuscript, a paper describing *PTTH* knockout line was published^32^.)

In the present study, to tackle these issues, we first evaluated and confirmed effectiveness of transcriptional and translational enhancers in *B. mori*. Using improved UAS vectors in which these enhancers are exploited, we generated strains that strongly express membrane-targeted GFP, calcium indicator protein GCaMP5G, and tetanus toxin light chain (TeTxLC) which blocks synaptic transmission, under the control of cell type-specific GAL4 drivers. Enhanced protein expression made it possible to visualize neural morphology and activity with drastic improvements. In addition, TeTxLC was useful to silence neural transmission of interest, such as BmOR1-, PTTH-, and bombyxin-expressing neurons. We addressed *in vivo* functions of PTTH and bombyxin in *B. mori*, and suggested that these hormones are important but not necessary for development and growth. Furthermore, we unexpectedly found that actions of PTTH and bombyxin on larval development are sex-differential.

## Results

### Enhancement of GFP expression by transcriptional and translational enhancers and improvements in visualization of neural projection patterns

**Figure 1:**
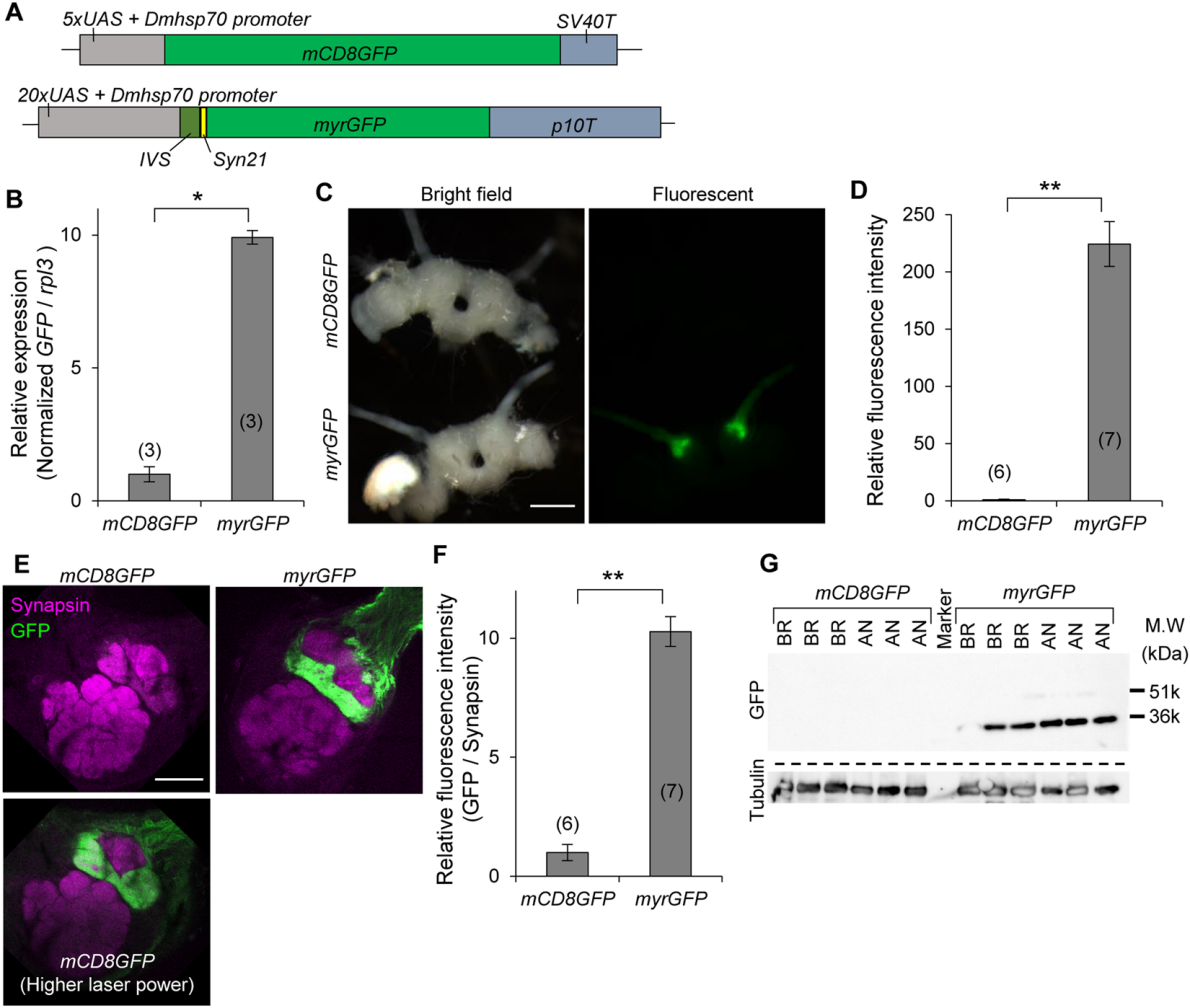
Enhancement of protein expression by refinement of UAS vector. (**A**) Structure of UAS vectors used for GFP expression in the present study. Upper panel indicates conventional UAS vector used to generate *UAS-mCD8GFP* strain previously. Lower panel indicates improved vector for *UAS-myrGFP* strain, which has increased copies of the GAL4 binding sites (*20xUAS*), an intervening sequence derived from *myosin heavy chain* gene of *Drosophila melanogaster* (*IVS*), synthetic translational enhancer (*Syn21*), and 3′UTR of AcNPV *p10* gene (*p10T*). mCD8 (mouse CD8) and myr (*N*-myristoylation) sequences are membrane targeting signals. (**B**) Relative expression levels of GFP mRNA in the male antennae determined by qRT-PCR. *UAS-mCD8GFP* and *UAS-myrGFP* strains were crossed to *BmOR1-GAL4* driver, which expresses GAL4 in the major sex pheromone receptor (BmOR1)-expressing cells. (**C**) Bright field and fluorescent images of dissected male brains under an epifluorescent microscope. Bright GFP signal was observed in the antennal nerves and antennal lobes of the myrGFP-expressed moth, whereas GFP fluorescence was barely observed in those of the mCD8GFP-expressed moth. (**D**) Quantification of GFP fluorescence intensities in the antennal lobes. (**E**) Confocal images of immunostained antennal lobes. Images of upper panels were taken with the same laser power. GFP signal could be observed with a higher laser power in the mCD8GFP-expressed sample (lower panel). (**F**) Relative fluorescent intensities of GFP in toroid, where BmOR1-expressing cells input. (**G**) Western blot analyses of GFP and α-tubulin of the brains (BR) and antennae (AN). Expected sizes of mCD8GFP and myrGFP are 51 and 36 kDa, respectively. Under the same exposure time, signal was detected only in the myrGFP-expressed samples. *P < 0.0001, **P < 0.00001, *t*-test. Number of samples are indicated in the parentheses. Bars, 500 μm (**C**) and 100 μm (**E**).

**Figure 2:**
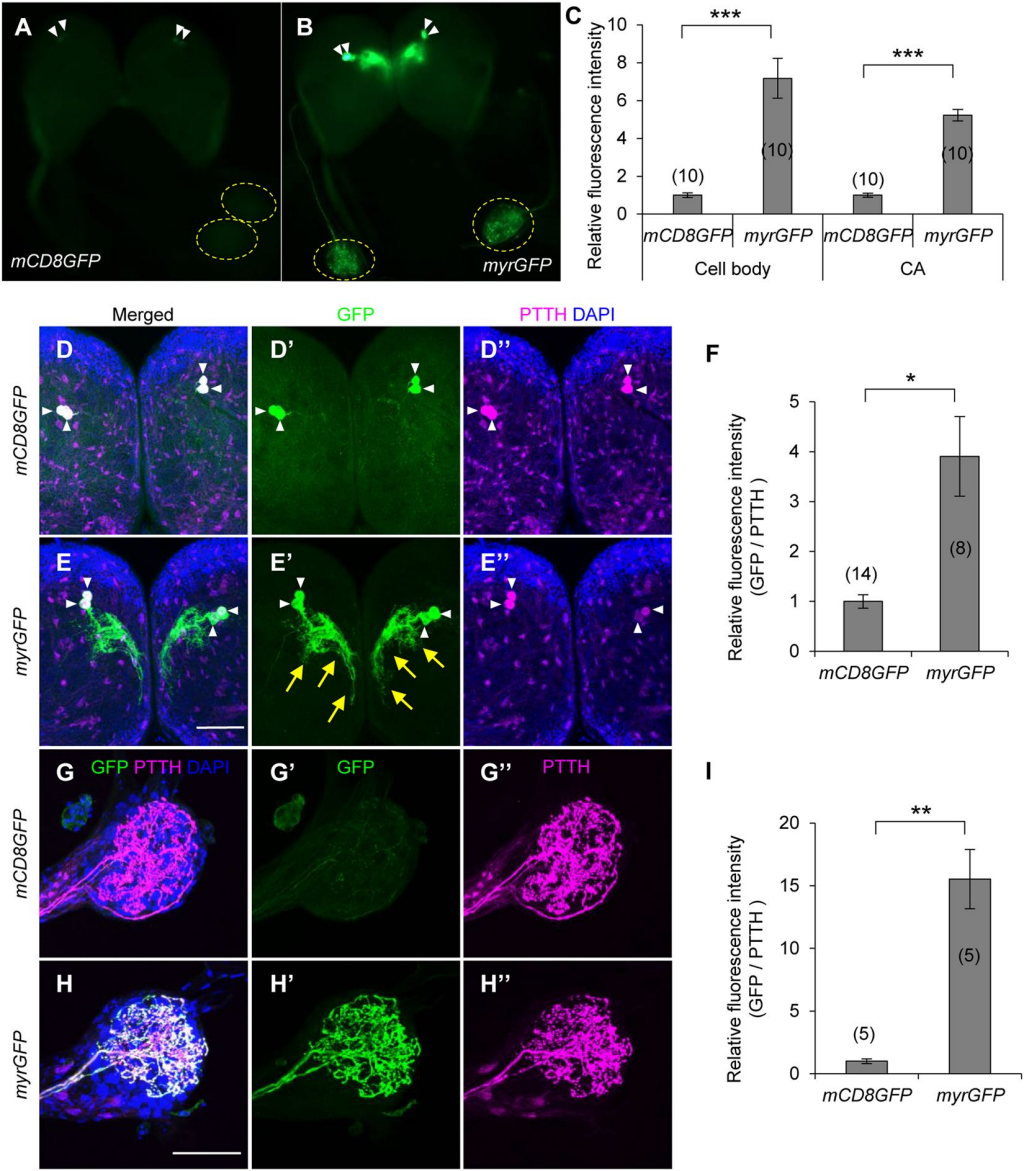
Improved visualization of projection pattern of PTTH neurons. (**A**,**B**) Fluorescent images of wandering larval brains under an epifluorescent microscope. mCD8GFP (**A**) or myrGFP (**B**) were expressed by *PTTH-GAL4* strain. White arrowheads and yellow dotted circles indicate cell bodies and corpora allata (CA), respectively. (**C**) Quantification of GFP fluorescence intensities in the cell bodies and CA. (**D**,**E**,**G**,**H**) Confocal images of immunostained brains (**D**,**E**) and CA (**G**,**H**). White arrowheads and yellow arrows indicate cell bodies and neurites, respectively. Magenta signals outside PTTH neurons are background DsRed expression driven by selection marker (3xP3-DsRed). (**F**,**I**) Relative fluorescence intensities of GFP in the cell bodies (**F**) and CA (**I**). *P < 0.01, **P < 0.005, ***P < 0.0002, *t*-test. Bars, 500 μm (**A**) and 100 μm (**E,H**). Number of samples are indicated in the parentheses.

**Figure 3:**
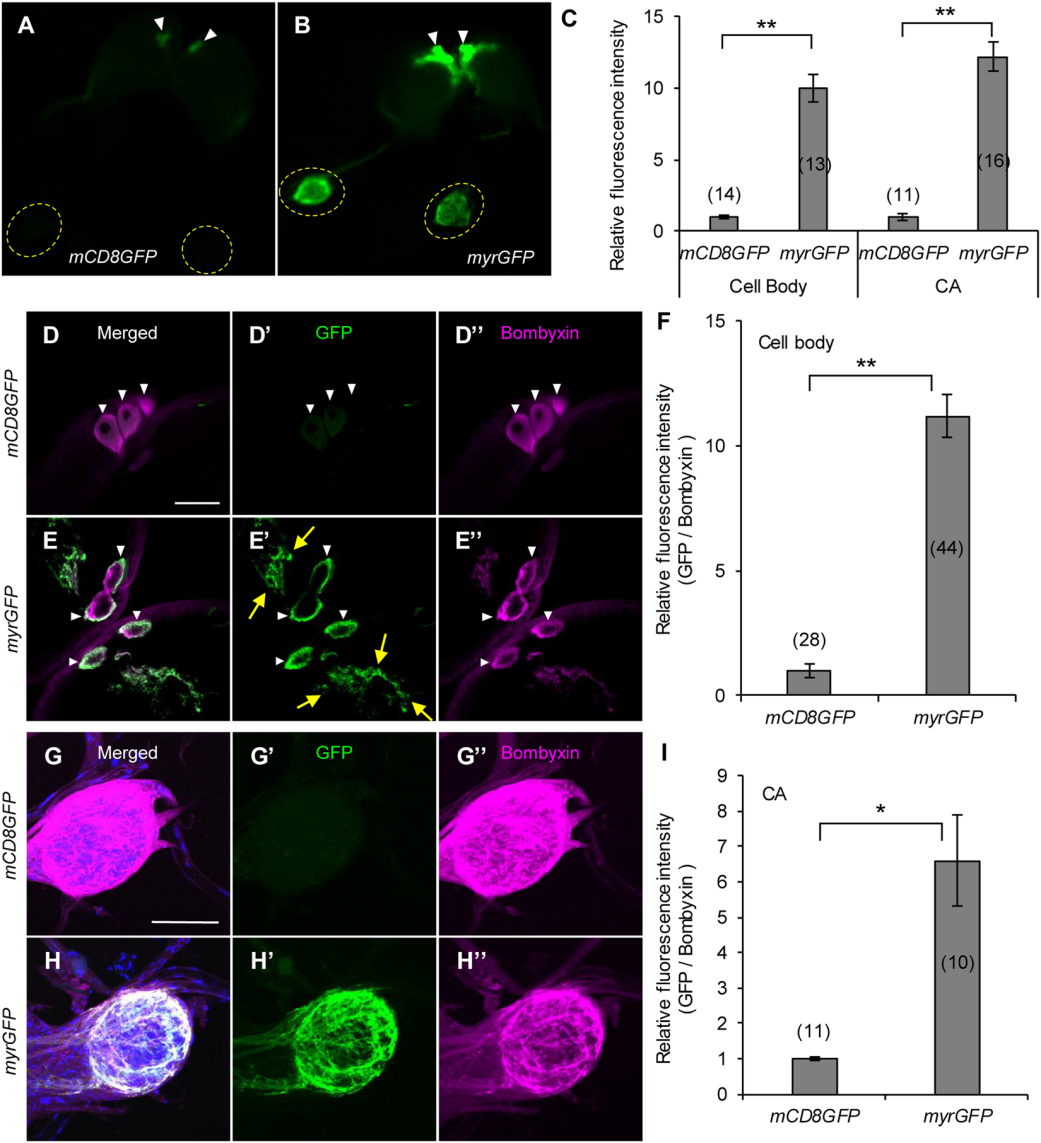
Improved GFP expression in bombyxin neurons. (**A**,**B**) Fluorescent images of final instar larval brains under an epifluorescent microscope. mCD8GFP (**A)** or myrGFP (**B**) were driven by *bombyxin-GAL4*. White arrowheads and yellow dotted circles indicate cell body clusters and CA, respectively. (**C**) Quantification of GFP fluorescence intensities in the cell bodies and CA. (**D,E,G,H**) Confocal images of immunostained brains (**D,E**) and CA (**G,H**). White arrowheads and yellow arrows indicate cell bodies and neurites, respectively. Only two to three cells are visible, since pictures of one optical section of confocal observation are shown (**D,E**). (**F,I**) Relative fluorescence intensities of GFP in the cell bodies (**F**) and CA (**I**). *P < 0.002, **P < 0.0001, *t*-test. Bars, 500 μm (**A**) and 100 μm (**E**,**H**). Number of samples are indicated in the parentheses.

To increase the efficiency of GAL4/UAS system in silkmoths, we constructed an improved *UAS-GFP* transformation vector by modifying the conventional *UAS-mCD8GFP* vector^8^. Based on the refinements reported in *D. melanogaster* ^12,13^, we increased *UAS* copy number and inserted intervening sequence (*IVS*), synthetic translational enhancer (*Syn21*), and terminator of *Autographa californica nuclear polyhedrosis virus* (*AcNPV*) *p10* gene *(p10T*), as transcriptional and translational enhancers (Fig. 1A). In addition, to enhance membrane-targeting efficiency, we used *N*-myristoylation signal (*myr*) instead of mouse CD8 sequence. Efficiency of germline transformation was not different between the novel and conventional vectors (data not shown), suggesting that addition of these enhancers does not cause prominent cytotoxicity. We crossed the two *UAS-GFP* lines to three *GAL4* strains characterized previously [*BmOR1-GAL4* (Fig. 1), *PTTH-GAL4* (Fig. 2), and *bombyxin-GAL4* (Fig. 3)]^5,7^, and compared their GFP expression. In the process of rearing, there was no obvious retardation or arrest of development, again suggesting that use of the enhancer elements does not have negative effects on *B. mori* development.

First, we found that the level of GFP mRNA expression in the antennae was increased by 10-fold in *UAS-myrGFP* line compared to that in *UAS-mCD8GFP* line, in combination with *BmOR1-GAL4* driver (Fig. 1B). Next, we evaluated improvement in the level of protein expression. Although GFP fluorescence was barely visible in the mCD8GFP-expressing moths under an epifluorescent microscope, bright signals were observed in the antennal nerves and lobes of myrGFP-expressing moths (Fig. 1C and D: more than 200-fold increase in intensity). Immunostaining also confirmed the enhancement of GFP protein in myrGFP-expressing animals (Fig. 1E and F). As previously reported, axon terminals of myrGFP-expressing cells converged onto toroid, indicating that the enhanced GFP expression did not deteriorate proper axon targeting. Western blot analyses showed that the level of GFP protein expression is drastically enhanced in *UAS-myrGFP* strain (Fig. 1G). These results collectively indicate that transcriptional and translational enhancers additively and/or synergistically upregulated GFP expression.

Further, to compare GFP expression at single-cell level, we used *PTTH-GAL4* (Fig. 2) and *bombyxin-GAL4* (Fig. 3) strains, which express GAL4 in two and four neurons per hemisphere, respectively. In both cases, higher GFP fluorescence in the cell bodies and neural processes were observed in myrGFP-expressing animals (Figs 2A–C and 3A–C). Especially in the corpora allata (CA), where axons of PTTH- and bombyxin-expressing neurons terminate, GFP signal was clearly detectable in myrGFP-expressing larvae whereas that of mCD8GFP-expressing animals was almost invisible. Signals of GFP was observed only in the PTTH or bombyxin neurons, indicating that increased GFP expression does not induce undesired ectopic expression. Immunostaining with internal control further confirmed improvements in protein expression and visualization of neural projection patterns (Figs 2D–I, 3D–I, and Supplementary Fig. S1). These results indicate that the newly constructed *myrGFP* transgene drastically enhances GFP expression in neurons and enables analyses at single-cell resolution in *B. mori*.

### Neural activity imaging with GCaMP5G

**Figure 4:**
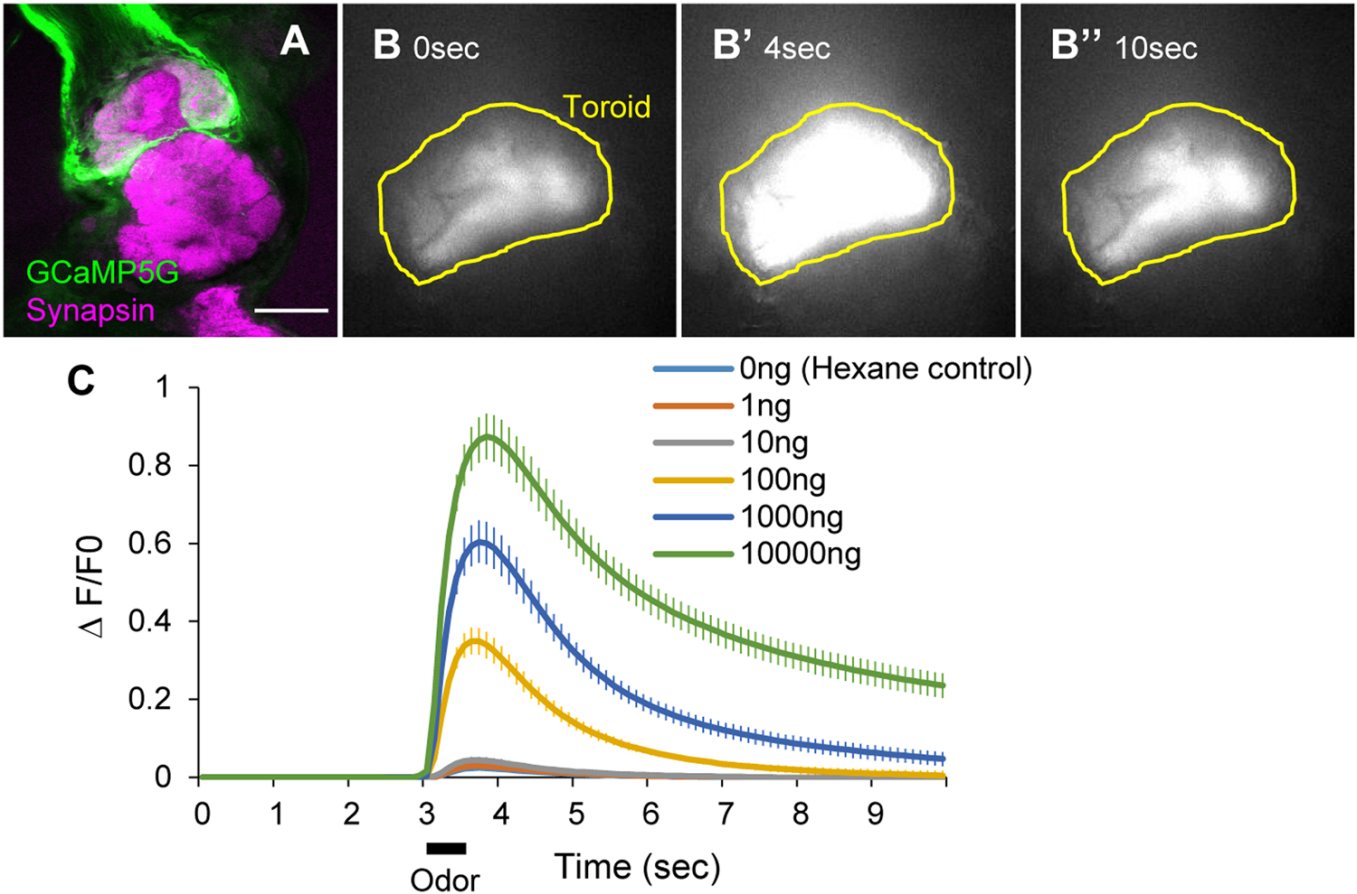
Visualization of neural activity by enhanced GCaMP5G expression. (**A**) Confocal image of the immunostained antennal lobe of male moth (*BmOR1* > *GCaMP5G*). (**B**) Time course of GCaMP fluorescence change in response to 10 μg bombykol stimulation. (**C**) Normalized dose-response relationship between fluorescence change and amount of bombykol used for stimulation. Bar, 100 μm. N = 10.

Having confirmed that our improved vector enhances transgene expression in silkmoths, we next generated an *UAS-GCaMP5G* strain for neural activity imaging (Fig. 4)^33^. Although *UAS-GCaMP2* strain was generated previously^34^, we aimed to improve the sensitivity of Ca^2+^ imaging using the highly optimized calcium indicator in combination with enhanced protein expression. We evaluated these points by expressing GCaMP5G in BmOR1-expressing cells. The GCaMP5G-expressing cells correctly converged axons onto toroid (Fig. 4A). Bombykol stimulation caused increase in fluorescence intensity in toroid (Fig. 4B and Supplementary video S1) in a dose-dependent manner, with higher ΔF/F ratio in GCaMP5G than that observed in GCaMP2 [ca. 10% (GCaMP2) vs 60% (GCaMP5G) in response to 1,000 ng stimulation] (Fig. 4C). These results indicate that use of optimized calcium indicator and enhancement of protein expression improve sensitivity of neural activity imaging.

### Generation and verification of UAS-TeTxLC strain that can block synaptic transmission

**Figure 5:**
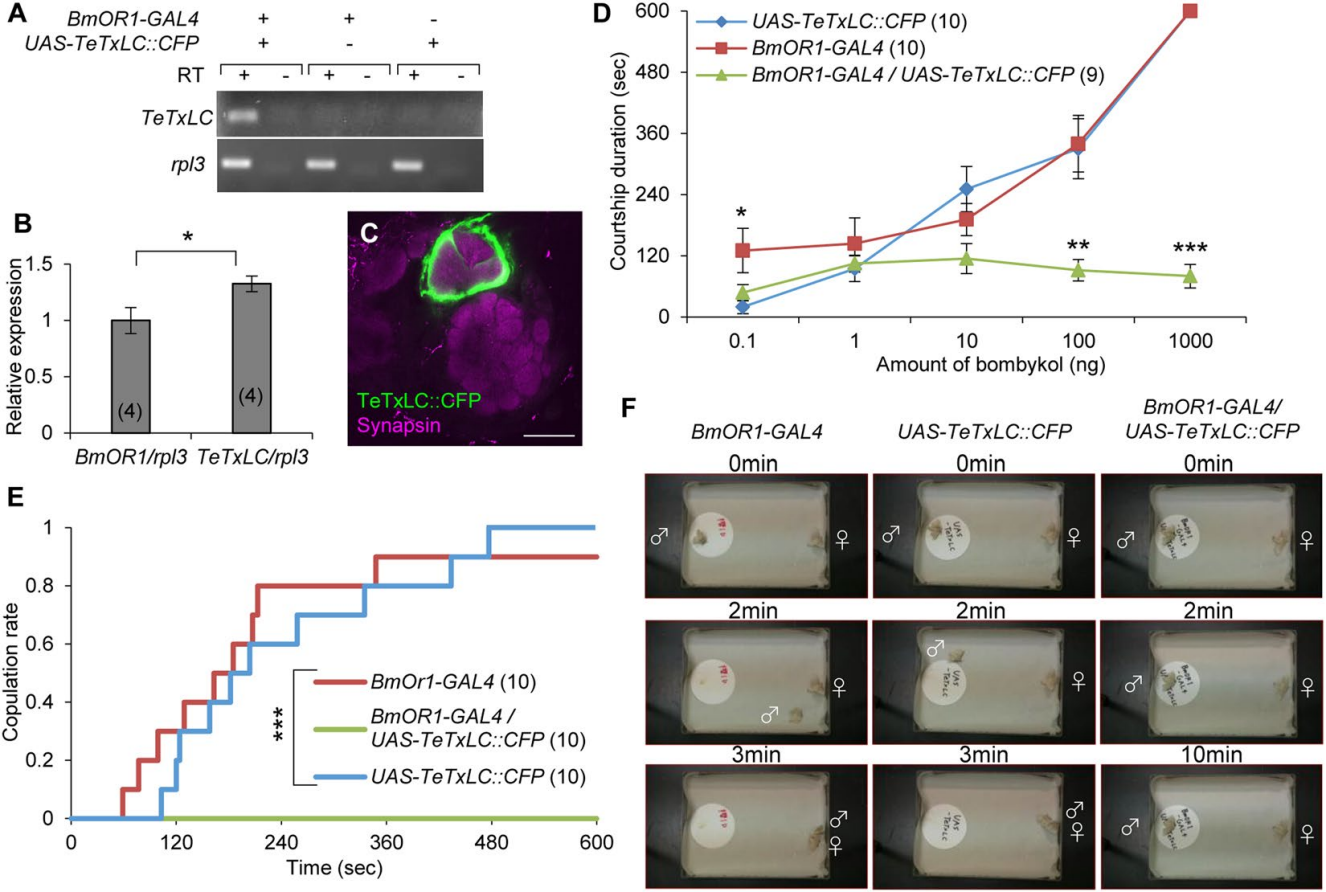
Blockade of neural transmission in BmOR1-expressing cells by TeTxLC. (**A**) RT-PCR analysis of *TeTxLC* expression in the male antennae. RT, reverse transcription. No genomic contamination was confirmed by RT- experiments. (**B**) Expression level of *TeTxLC* was significantly higher than that of endogenous sex pheromone receptor (*BmOR1*) revealed by qRT-PCR. *P < 0.05, *U*-test. (**C**) Confocal image of immunostained antennal lobe. TeTxLC expression did not disturb proper axon targeting. Bar, 100 μm. (**D**) Dose-response relationship of courtship duration and amount of bombykol used for stimulation. Wing-flapping time during 10 min observation period was measured. *P < 0.05, **P < 0.01, ***P < 0.005, *Tukey-Kramer’*s HSD test after ANOVA. (**E**) Cumulative copulation rate against observation time. ***P < 0.0001, log-rank test. (**F**) Still images of behavior movies. Number of samples are indicated in the parentheses.

For analysis of neural circuit functions, it is essential to have genetic tools that can activate or inhibit neurons of interest. We previously succeeded to artificially control neural activity in *B. mori* using the *UAS-dTrpA1* strain^8^ that expresses a warmth-sensor channel of *D. melanogaster*, but failed to inhibit neural transmission using the *UAS-Shibire* ^*ts1*^ strain that expresses a temperature-sensitive mutant of dynamin, for unknown reasons. In the present study, we focused on TeTxLC, which prevents neurotransmitter release by cleaving synaptobrevin, an essential synaptic protein for exocytosis^35^. TeTxLC is successfully used to block neural transmission in a variety of animals. After generating *UAS-TeTxLC::CFP* strains using our improved vector backbone, we first examined its effectiveness by crossing the strain to *Actin A3-GAL4* driver (Supplementary Table S1). No larva hatched by ubiquitous TeTxLC expression, suggesting that TeTxLC functions in *B. mori*. Next, we expressed TeTxLC in BmOR1-expressing cells and evaluated its effects on behavior (Fig. 5). Expression level of *TeTxLC* mRNA in the antennae was higher than that of *BmOR1* (Fig. 5B). Compared with the previous study using the same GAL4 strain, where expression level of transgene mRNA was approximately 10% of *BmOR1* ^5^, these results again confirmed enhanced mRNA expression by our improved vector. Axons of TeTxLC-expressing cells correctly targeted toroid (Fig. 5C), indicating that inhibition of synaptic transmission does not affect proper glomerular targeting. TeTxLC expression in BmOR1-expressing cells greatly reduced behavioral response of males upon stimulation with high concentrations of bombykol (100–1,000 ng: Fig. 5D). Similar results were obtained in experiments using another *UAS-TeTxLC* strain (Line #2: data not shown). *BmOR1-GAL4* strain expresses GAL4 in most but not all BmOR1-expressing cells (96.8%)^6^. Since a large number of antennal cells express BmOR1^17,18^, many BmOR1-expressing cells escape from blockade by TeTxLC (estimated to be at least more than 500 cells). Thus, residual behavioral response to bombykol could be explained by activities of cells that did not express TeTxLC. Finally, we investigated functional importance of BmOR1 neural transmission in copulation success (Fig. 5E and F). Male moths whose BmOR1 neural transmission was blocked by TeTxLC did not show courtship behavior to virgin female at all, although almost all control moths started courtship behavior upon detection of a female and succeed in copulation within 10 min (Supplementary video S2–S4). These results were consistent with the previous study where *BmOR1* mutants were reported to show no courtship behavior, further confirming the effectiveness of TeTxLC expression in *B. mori* ^19^.

### PTTH neural transmission regulates developmental timing and body weight, but is not necessary for molting and metamorphosis

**Figure 6:**
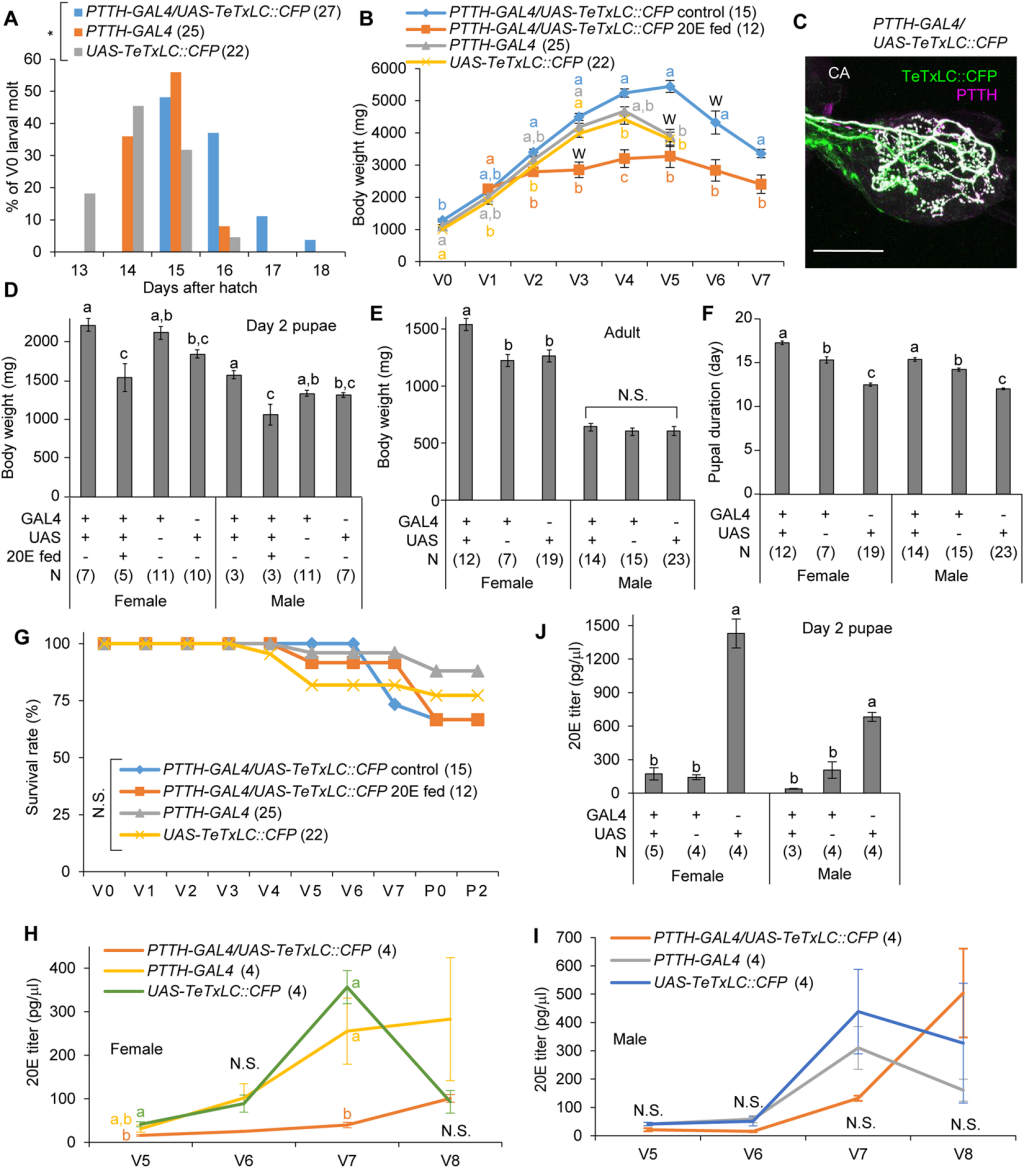
Blockade of PTTH neural transmission delays developmental timing. (**A**) Timing of V0 larval molting. *P < 0.00005, Chi-square test. (**B**) Developmental change in larval body weights. W indicates the day when majority of the larvae started wandering. (**C**) Confocal image of immunostaining in CA. (**D,E**) Body weights in day 2 pupae (**D**) and adult moths (**E**). (**F**) Pupal duration. (**G**) Survival rate during larva-to-pupa development. (**H**–**J**) 20E titer determined by ELISA in female larvae **(H**), male larvae (**I**), and pupae (**J**). Statistically different groups are shown in different characters (*P* < 0.05, *Tukey-Kramer’*s HSD test after ANOVA). N.S., not significant (*P* > 0.05). Bar, 100 μm. Number of samples are indicated in the parentheses.

We next addressed the importance of PTTH neural transmission on *B. mori* development (Fig. 6). Using the *PTTH-GAL4* driver, we generated *PTTH-GAL4/UAS-TeTxLC::CFP* animals and confirmed that TeTxLC is transported to all synaptic boutons in the CA (Fig. 6C). Blockade of PTTH neural transmission did not arrest larval or pupal development. No larvae showed extra- or precocious-molting. However, severe developmental phenotypes were observed in later stages of development, and we thus concentrated our detailed analyses on the last instar larvae (fifth-instar in *B. mori*), pupae, and adults. TeTxLC expression in PTTH neurons significantly delayed the onset of 4th-to-5th larval molting (Fig. 6A and Supplementary Fig. 2), shifted the onset of wandering (Fig. 6B), and increased the maximal larval body weight (Fig. 6B and Supplementary Fig. 2). Since the rates of body weight gain were similar among the control and TeTxLC-expressing animals, increase in maximal body weight can be attributed to the extended duration of growth period. Pupal body weight of *PTTH-GAL4/UAS-TeTxLC::CFP* line was significantly higher than that of *UAS-TeTxLC::CFP* line, but not than that of *PTTH-GAL4* line (Fig. 6D and Supplementary Fig. 2). The increased body weight and developmental delay observed in *PTTH-GAL4/UAS-TeTxLC::CFP* line were reversed by 20E-feeding (Fig. 6B and D), suggesting that the reduction of 20E titer by suppression of PTTH neural transmission is the cause of these phenotypes. Expression of transgenes and 20E-feeding did not affect survival rates (Fig. 6G and Supplementary Fig. 2). Remarkably, suppression of PTTH neural transmission delayed but did not abolish the onset of 20E titer rise (Fig. 6H and I), which occurs before larval-to-pupal molting^36^, suggesting that PTTH neural transmission regulates proper timing of 20E increase but is not essential for ecdysone biosynthesis.

Pupal duration was the longest in *PTTH-GAL4/UAS-TeTxLC::CFP* line, followed by *PTTH-GAL4* and *UAS-TeTxLC::CFP* lines (Fig. 6F). In day 2 pupae, 20E titers of *PTTH-GAL4/UAS-TeTxLC::CFP* and *PTTH-GAL4* strains were significantly lower than that of *UAS-TeTxLC::CFP* strain (Fig. 6J). These results suggest that reduction of PTTH attenuated 20E titer increase in the initial stage of pupae, where a huge PTTH surge and a following 20E surge occur^36^.

Interestingly, in adults, significant increase in body weight by PTTH silencing was observed only in females, indicating sexual dimorphism in the roles of PTTH in adults (Fig. 6E). Adults of *PTTH-GAL4/UAS-TeTxLC::CFP* line showed normal courtship behavior and laid normal number of eggs, which hatched normally (data not shown), suggesting that blockade of PTTH neural transmission have no effects on reproduction.

There was an overall tendency that *PTTH-GAL4* line had higher body weight and slower development than *UAS-TeTxLC::CFP* line, although these lines had been backcrossed multiple times and are thought to share the same genetic background. Since PTTH expression is regulated by cis-regulatory elements in the promoter region^37^, which is also used to express GAL4 in the *PTTH-GAL4* line^7^, PTTH expression may be decreased in *PTTH-GAL4* line by competition of regulatory factors. Otherwise, it is possible that cytotoxicity of GAL4 partly disturbed PTTH neural functions.

Taken together, these results indicate that PTTH neural transmission plays important roles in regulation of developmental timing and body weight through control of 20E biosynthesis, but is not necessary for molting, metamorphosis, and survival. Targeted expression of TeTxLC in PTTH neurons caused much milder phenotypes compared to those of the *PTTH* loss-of-function mutants^32^. These differences may be attributed to incomplete suppression of PTTH neurotransmission, as TeTxLC efficiently blocks evoked release but not spontaneous miniature transmitter release^35^. Also, since PTTH is expressed not only in the brains but also in the gut and epidermis^38^, it is possible that gut and epidermis-expressed PTTH compensated the semi-lethal phenotypes observed in the *PTTH* mutants.

### Bombyxin neural transmission regulates growth rate but not growth period

**Figure 7:**
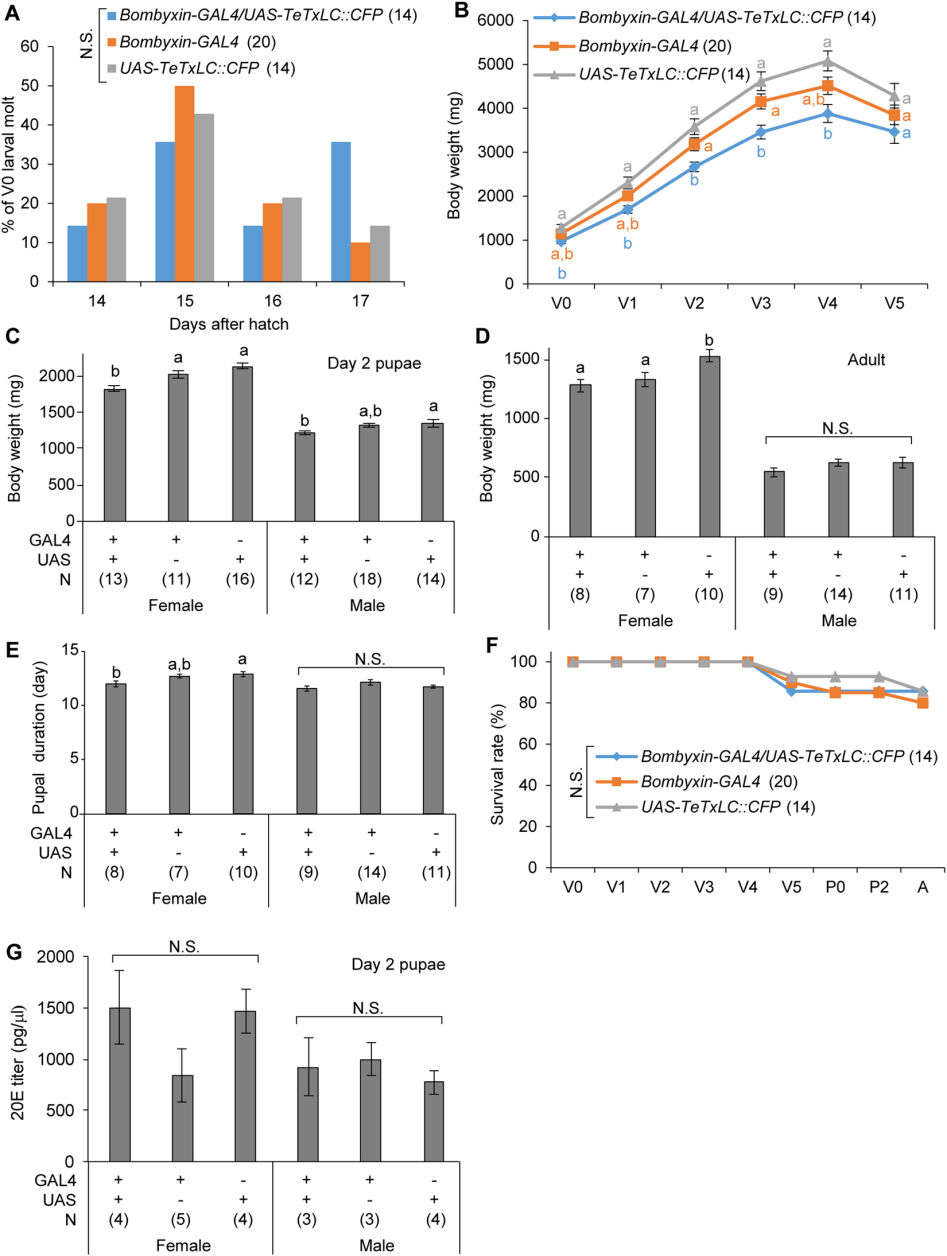
Blockade of bombyxin neural transmission decreases larval growth rate. (**A**) Timing of V0 larval molting. (**B**) Developmental change in larval body weights. (**C**,**D**) Body weights of day 2 pupae (**C**) and adult moths (**D**). (**E**) Pupal duration. (**F**) Survival rate during larva-to-pupa development. (**G**) 20E titer in day 2 pupae. Statistically different groups are shown in different characters (*P* < 0.05, *Tukey-Kramer’*s HSD test after ANOVA). N.S., not significant (*P* > 0.05). Number of samples are indicated in the parentheses.

In *D. melanogaster*, growth rate and body size are regulated by antagonistic actions of insulin and ecdysone signaling^21–25^. To gain insights into *in vivo* function of insulin signaling on *B. mori* development, we expressed TeTxLC in bombyxin neurons, which are one of major sources of insulin-like peptides in larvae^30,39^. Blockade of bombyxin neural transmission did not delay larval development, but significantly reduced body weight from larvae to adult, and slightly shortened pupal duration (Fig. 7A and E). Some of these phenotypes were observed only in females, suggesting sexual differences in roles of insulin signaling. In a similar manner to *PTTH-GAL4* line, *bombyxin-GAL4* line tended to show mild phenotypes of what we observed in *bombyxin-GAL4/UAS-TeTxLC::CFP* line, implying unexpected side effects of promoter used for GAL4 expression or the cytotoxicity of GAL4. No significant difference in survival rate among lines was observed (Fig. 7F). In addition, there was no significant difference in 20E titer among strains in day 2 pupae, when 20E titer is the highest (Fig. 7G), suggesting that silencing of bombyxin neurons did not affect ecdysone biosynthesis. Adults whose bombyxin neural transmission was blocked normally copulated and laid eggs (data not shown). These results suggest that bombyxin neural transmission plays important roles in control of growth rate but not the duration of growth period, and are dispensable for molting, metamorphosis, and survival.

### PTTH and bombyxin signaling antagonistically and sex-differentially regulate larval development

**Figure 8:**
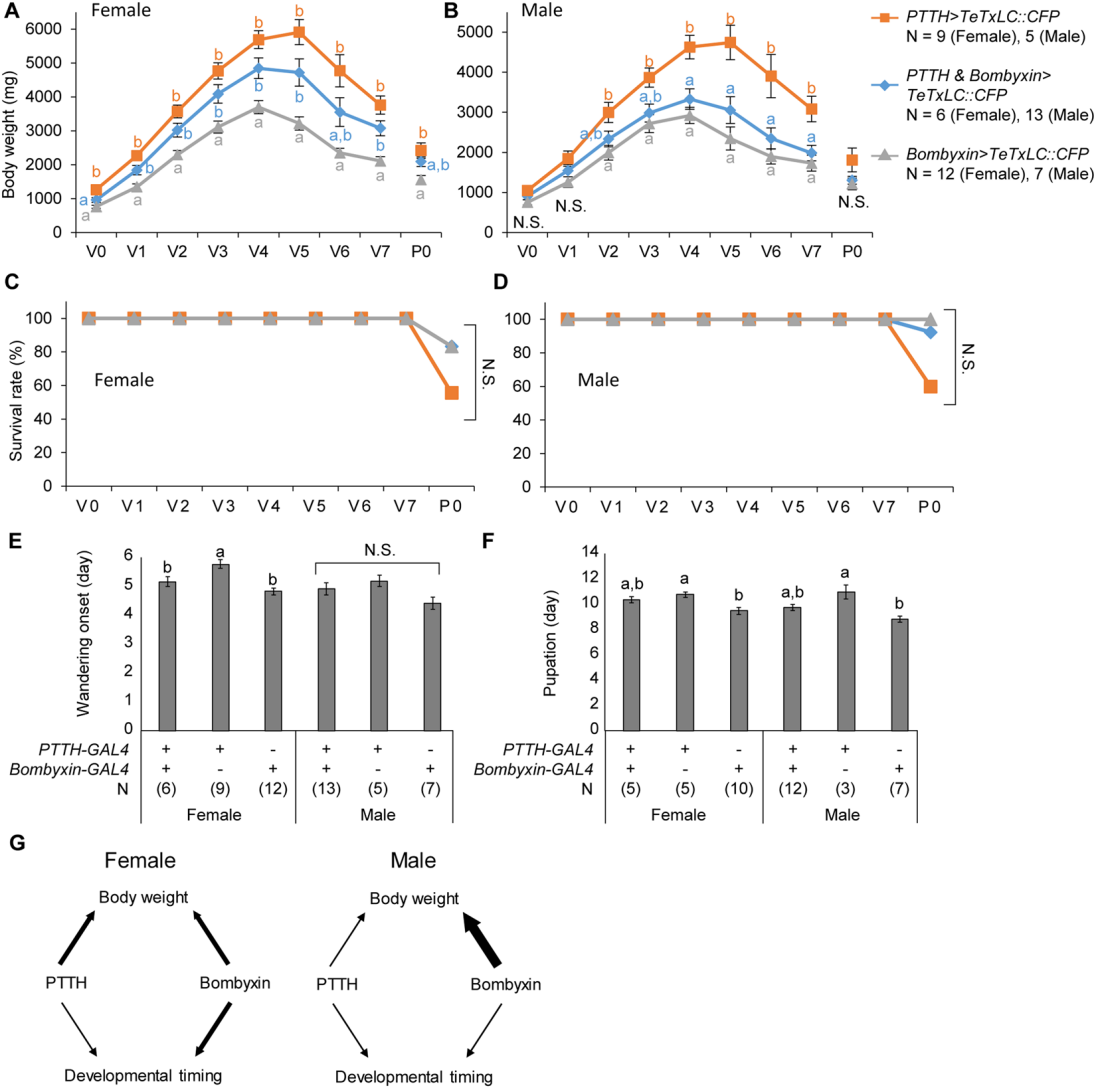
Sex differential actions of PTTH and bombyxin signaling on larval development. (**A**,**B**) Developmental change of body weights in females (**A**) and males (**B**). (**C**,**D**) Survival rate during larva-to-pupa development in females (**C**) and males (**D**). (**E**,**F**) Timing of wandering (**E**) and pupation (**F**) onset. Days from start of fifth instar is shown. Statistically different groups are shown in different characters (*P* < 0.05, *Tukey-Kramer’*s HSD test after ANOVA). N.S., not significant (*P* > 0.05). Number of samples are indicated in the parentheses. (**G**) A model of sexually different regulation of body weight and developmental timing by PTTH and bombyxin in larvae.

Neural silencing experiments showed that PTTH and bombyxin signaling play distinctive roles in larval development, and has opposite effects on body weight. In addition, there were sexual differences in actions of these signaling. These results prompted us to investigate which signaling has stronger effects on development and whether there are sexual differences in the actions of these pathways. To address these questions, we blocked neural transmission of PTTH, bombyxin, or both PTTH and bombyxin neurons by TeTxLC and analyzed larval-to-pupal development (Fig. 8A and B). There were sex-differential effects of PTTH and bombyxin signaling on development. Silencing of both PTTH and bombyxin neurons caused a growth phenotype similar to PTTH neuron silencing in females, but to bombyxin neuron silencing in males. These results suggest that PTTH and bombyxin signaling have dominant function on larval development in females and males, respectively. Intriguingly, simultaneous silencing of PTTH and bombyxin neural transmission did not cause developmental arrest or mortality in larvae, and survival rate was not significantly different among strains (Fig. 8C and D). In contrast to larval growth phenotypes, the onset of wandering was similar to that of bombyxin neuron silencing in females, but no significant difference was observed in males (Fig. 8E). In addition, the onset of pupation phenotype caused by silencing of both PTTH and bombyxin was intermediate between PTTH and bombyxin neuron silencing in both males and females (Fig. 8F). These results suggest that developmental timing is dominantly regulated by bombyxin signaling in females, but not in males. Taken together, these findings indicate that growth and developmental timing are antagonistically and sex-differentially regulated by PTTH and bombyxin (Fig. 8G).

## Discussion

In the present study, we refined *in vivo* heterologous protein expression in *B. mori*, utilizing transcriptional and translational enhancers in the GAL4/UAS system. Our approaches did not induce any obvious negative effects on development and physiology, indicating that these enhancers are useful for generating silkmoth strains with high levels of transgene expression. Thus, the present refinement will benefit not only basic science but also applied studies that use silkmoths. Enhanced GFP expression improved visualization of neural projection patterns and enabled detailed analyses of neural shape at single-cell resolution. In addition, enhanced expression of GCaMP5G made it possible to measure neural activity with higher brightness and sensitivity. Utilizing this refined system, we established methods to block neural transmission by expression of TeTxLC. Ectopic expression of TeTxLC successfully inhibited larval hatch as well as behavioral response to sex pheromone in adult males. Furthermore, we uncovered the functional importance of PTTH and bombyxin signaling on development. Blockade of these neural transmission impaired developmental timing or growth rate, but did not arrest development or cause mortality. These phenotypes are similar to those observed in the recent studies in *D. melanogaster* ^21,25,40^, suggesting that central mechanisms of postembryonic development and growth are evolutionally conserved between Lepidoptera and Diptera. In contrast, differences in phenotypes were also observed, which may reflect divergent evolutionary histories between these species. In addition, the present study revealed that larval development is sex-differentially regulated by PTTH and bombyxin signaling.

Historically, PTTH and bombyxin were isolated from brains of *B. mori* after long-term efforts to purify the ‘brain hormone’, which promotes eclosion of brain-ablated pupae of Eri-silkmoth, *Samia ricini*, and J122 x C115 strain of *B.mori* ^26,29^. Although it has long been known that PTTH stimulates the PGs to produce ecdysone and bombyxin promotes blood sugar uptake and growth in *B. mori* ^28,30,36^, their necessity *in vivo* was not addressed until recently. The present study provides evidence that PTTH and bombyxin neural transmission play important roles in regulation of the growth period and growth rate.

To date, several lines of evidence have suggested that PTTH is not essential for molting and metamorphosis in *B. mori*. First, long-term storage of brain-ablated silkmoth pupae, which were used for PTTH bioassay, resulted in spontaneous eclosion^41^, indicating that brain-derived factor is not necessary for the PG activation in pupae. Second, recent studies in *B. mori* revealed that ecdysteroid biosynthetic activity of the PGs is regulated not only by PTTH but also by multiple tropic and static factors^41^. Third, in *D. melanogaster*, PTTH cell ablation or perturbation of PTTH receptor (*torso*) and its downstream pathways in the PGs significantly prolonged larval period but did not cause developmental arrest^40,42^. Furthermore, while we were preparing this manuscript, *PTTH* loss-of-function mutant lines were established and the study indicated that PTTH is not a necessary factor for molting and metamorphosis^32^. Thus, our present study provides further evidence that PTTH is one of important factors that regulate precise timing of the PG activation rather than a definitive factor for postembryonic development in *B. mori*.

Since the PG activity of *D. melanogaster* is regulated by various factors in a manner similar to *B. mori* ^22,23,43–46^, central mechanisms that PG activity is regulated by multiple factors to coordinate environmental and internal information may be evolutionally conserved among holometabolous insects. In contrast, we observed differences in detailed phenotypes between silkmoth and fly. Notably, loss of PTTH neurons causes prominent delay of development preferentially in the last instar (third-instar) larvae but not in pupae in *D. melanogaster* ^40^, whereas silencing of PTTH neurons delayed development in both larval and pupal stages in *B. mori*. A similar phenotype was reported in recent *PTTH* mutant lines^32^. These results suggest that PTTH plays major roles in larval-to-pupal development in *D. melanogaster*, but in both larval-to-pupal and pupal-to-adult development in *B. mori*.

In *D. melanogaster*, PTTH neurons project their axons onto the PGs and regulate the PG activity by direct neural transmission^40^. In contrast, in *B. mori*, PTTH is secreted from the corpora allata and circulating humoral PTTH activates the PG^31^. In addition, the PG activity is repressed by direct innervation from the thoracic ganglion, as well as circulating static factors^41^, although such static factors are not identified in *D. melanogaster*. These differences in the PG regulation between these species may explain the differences in PTTH actions on development.

With neural silencing experiments, we also revealed that bombyxin neurons play important roles in controlling growth rate. Although actions of bombyxin on larval development and metabolism have been intensively analyzed^24,27,28,30^, our present study for the first time addressed *in vivo* importance of bombyxin synthesized in the brain on growth and development. *B. mori* has approximately 40 bombyxin genes in the genome^39^, whereas *D. melanogaster* has only eight insulin-like peptide (ILP) genes^24^. The increase in copy number of bombyxin genes in the genome suggests functional diversification of insulin signaling^39^, but makes genetic analyses difficult. Practically, it is technically challenging to address *in vivo* importance of bombyxin by knockout approaches. Thus, since majority of bombyxin genes are expressed in bombyxin neurons of the brain^24,39^, our approach provides important insights into *in vivo* function of bombyxin. Notably, bombyxin and ILPs are expressed in many tissues other than the brain in both *B. mori* and *D. melanogaster* ^24,47,48^. Recent studies in *D. melanogaster* revealed that these ILPs play distinct and essential roles in development, growth, and metabolism, indicating the importance of ILPs secreted from tissues other than the brain^24,49^. It will be interesting to investigate bombyxin functions outside the brain on development and metabolism in the future studies.

Interestingly, we found that the importance of PTTH and bombyxin on larval development is different between sexes (Fig. 8). Delayed onset of 20E titer increase was more prominent in females than in males, when transmission of PTTH neurons was blocked (Fig. 6H and I). These results suggest the possibility that not only PTTH and bombyxin but also other humoral factors have sex differential titers and play sex-differential roles in insect development. Thus, it will be interesting to reveal the functional importance of sex-differential actions of hormones during juvenile-to-adult transition in insects in in the future studies.

## Methods

### Generation and rearing of transgenic silkmoths

In all experiments, larvae were reared on artificial diet (Nihon Nosan Kougyo, Yokohama, Japan) at 25 °C under a 12-h light/12-h dark photoperiod cycle. In 20E feeding experiments, larvae were reared on slices of artificial diet (7-8 g/slice) soaked with 20 μl of 1 mM 20E (Sigma) dissolved in 4% EtOH (approximate final concentration is 62.5 μg/g). Body weights were weighed every day between 3 to 6 h after the start of light-phase. Sex was determined by checking larval or pupal genitalia. Under a normal condition, majority of pupation occurs at 9th-day of fifth instar (P0 = V9). However, the timing of pupation deviates among individuals (V8-V11). Therefore, measurement of survival rate and body weights ceased at V5 or V7. Transgenic silkmoth strains were generated as described previously^3,8^, using the hyperactive *piggyBac* transposase (hyPBase) instead of the conventional transposase^50^. More than two lines were generated for each transgenic strain and levels of transgene expression were not different among lines. All transgenic strains were backcrossed to white-eyed diapausing strain, *white*, for more than three generations^8^. *UAS* strains generated in the present study (*UAS-myrGFP*, *UAS-GCaMP5G*, and *UAS-TeTxLC::CFP*) will be available from National Bio-Resource Project (NBRP): Silkworms after publication. Possession of transgenes was confirmed by checking fluorescent selection markers during the 2nd or 3rd instar larvae. To generate the triple transgenic strain that possesses *PTTH-GAL4*, *bombyxin-GAL4*, and *UAS-TeTxLC::CFP*, *PTTH-GAL4/UAS-TeTxLC::CFP* and *bombyxin-GAL4/UAS-TeTxLC::CFP* were crossed and their progenies were used. Since both *PTTH-GAL4* and *bombyxin-GAL4* strains possess the same selection marker (3xP3-DsRed), all larvae were labeled with color markers, weighed every day, and genotyped at day 0 pupae.

### Vector construction

Detailed procedures for vector construction are shown in Supplementary Figures S3–S6. PrimeSTAR HS DNA Polymerase and In-Fusion HD cloning kit (Takara-Bio, Kyoto, Japan) were used for construction. Vectors will be distributed upon request.

### PCR (qRT-PCR and genotyping)

Quantitative RT-PCR was conducted as described previously^20^. In the experiments using two *GAL4* strains with the same selection marker (*PTTH-GAL4* and *bombyxin-GAL4*), genotyping was performed using KAPA2G Robust PCR kit (Kapa Biosystems, MA). Primers used are listed in Supplementary Table S2.

### Quantification of fluorescence intensities

Fluorescent images of brains or CAs were taken under epifluorescent microscope (M165FC, Leica, Germany). Quantification was conducted using imageJ. After fluorescent imaging, samples were subjected to immunostaining.

### Immunohistochemistry

Immunohistochemistry was conducted as described previously^8^. Antibodies used in the present study are as follows: Rabbit anti-GFP antibody (1/1,000; Invitrogen), mouse anti-Synapsin monoclonal antibody [1/100; Developmental Studies Hybridoma Bank (DSHB), Iowa City, IA], mouse anti-PTTH monoclonal antibody (1/200; gift from Akira Mizoguchi)^31^, Rabbit anti-Bombyxin-A antibody (1/200; gift from Hiroshi Kataoka), Goat anti-Rabbit Alexa488, and Goat anti-Mouse Cy3 (1:200; Jackson ImmunoResearch Laboratories). Images were taken using the confocal microscope LSM5 (Carl Zeiss, Germany). Quantification of fluorescence was performed using imageJ. The value of fluorescent intensity of GFP was divided by that of internal control (Synapsin, PTTH, or Bombyxin) in every picture. Then, all relative values were normalized by the average value in mCD8GFP strain. Therefore, in all cases, the relative values of mCD8GFP strain become 1.

### Western blotting

Western blotting was conducted as described previously^20^. Rabbit anti-GFP antibody (1/10,000; Invitrogen, CA) or mouse anti-α-tubulin monoclonal antibody (1/10,000; DSHB) were used for detection.

### Ca^2+^ imaging

Ca^2+^ imaging was performed as described previously^34^. Images were acquired using 20x water-immersion objective lens (NA: 0.5) every 100 ms (10 Hz) for 10 sec imaging period. Humidified clean air was continuously flown to the antennae of fixed moth whose brain was exposed. Determined amount of bombykol diluted in hexane was soaked in a piece of filter paper. For stimulation, the airstream was switched to pass through the glass tube containing the filter paper for 0.5 sec. Fluorescence in toroid was analysed.

### Behavioral analysis

For quantification of responsiveness to bombykol, each male moth isolated in a small closed plastic cup (ϕ101 mm, height 44 mm), where a filter paper (No2. ϕ70 mm, ADVANTEC, CA) was placed, was stimulated by loading determined amount of bombykol to the filter paper. Behaviours were video-recorded for 10 min from the start of pheromone stimulation. Courtship duration (wing-flapping time) was measured manually. In the copulation assay, male (transgenic) and female (*white*) moths were placed in the opposite sides of open plastic case (221 × 161 × 43 mm) and the behavior was video-recorded until copulation (max recording time: 10 min).

### Ecdysone titer measurements

Haemolymphs collected from larvae and pupae were subjected to enzyme-linked immunosorbent assay (ELISA) using 20E EIA kits (Cayman Chemical) according to the manufacturer’s protocol.

### Statistics

All data are indicated by mean ± standard error. Sample number is shown in parenthesis. Statistical analyses were performed using JMP7 (SAS) or Excel toukei. For comparison of two groups, *Student*’s or *Welch*’s *t*-test was used after *F*-test. When the data was not normally distributed, *U*-test was used. For multiple comparisons, ANOVA followed by *Tukey-Kramer*’s *post hoc* test was conducted.

## Electronic supplementary material

Video S1

Video S2

Video S3

Video S4

Supplement Figs

Table S1

Table S2
