# Supplementary material for: Refinement of ectopic protein expression through the GAL4/UAS system in Bombyx mori: application to behavioral and developmental studies
Source: Sci Rep. 2017 Sep 18;7:11795. doi: 10.1038/s41598-017-12102-2 (PMC5603595; doi:10.1038/s41598-017-12102-2)
Supplement: Supplementary file 5 — Supplement Figs [file 41598_2017_12102_MOESM5_ESM.pdf]

Supplementary information for: Refinement of ectopic protein expression through the GAL4/UAS system in Bombyx mori: application to behavioral and developmental studies

Chiho Hara[1+], Koudai Morishita[1+], Seika Takayanagi-Kiya[1], Akihisa Mikami[2], Keiro Uchino[3], Takeshi Sakurai[2], Ryohei Kanzaki[2], Hideki Sezutsu[3], Masafumi Iwami[1], and Taketoshi Kiya[1+*]

[1]Division of Life Sciences, Graduate School of Natural Science and Technology, Kanazawa University, Kakuma-machi, Kanazawa, Ishikawa, 920-1192, Japan

[2]Research Center for Advanced Science and Technology, The University of Tokyo, 4-6-1 Komaba, Meguro-ku, Tokyo 153-8904, Japan

[3]Transgenic Silkworm Research Unit, Institute of Agrobiological Sciences, National Agriculture and Food Research Organization, 1-2 Owashi, Tsukuba, Ibaraki 305-8634, Japan

[*] kiya@staff.kanazawa-u.ac.jp

[+]These authors contributed equally to this work

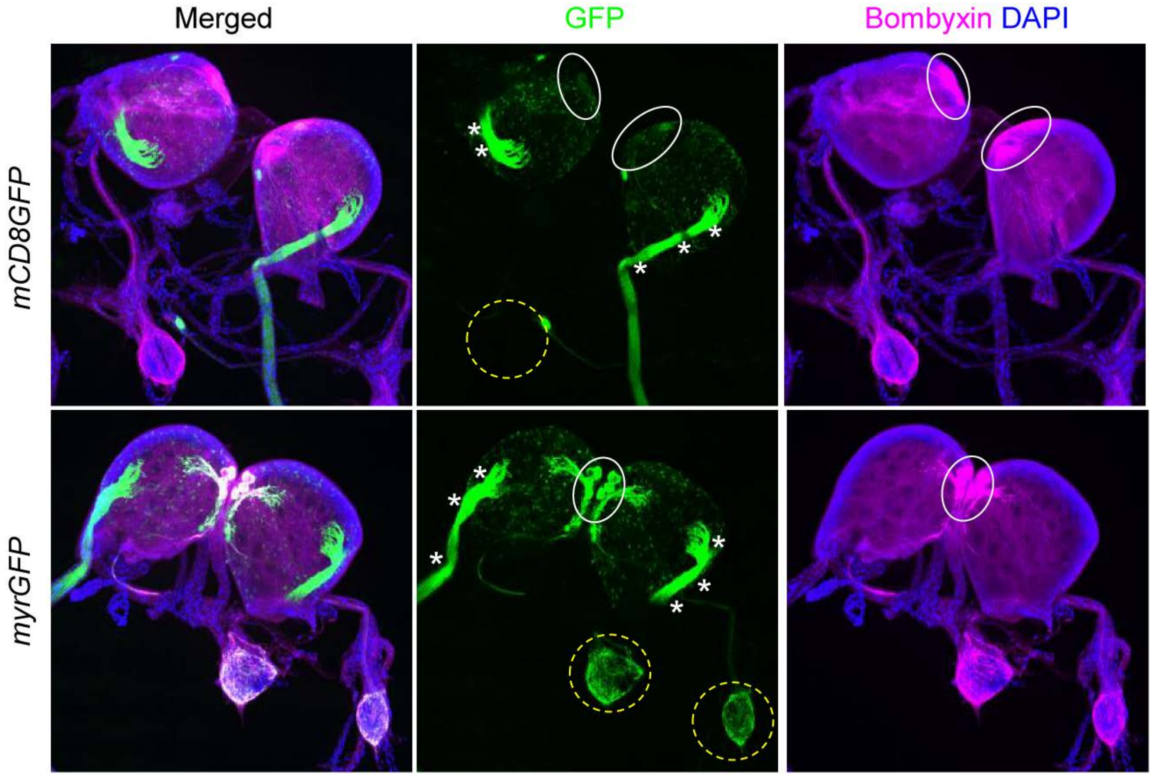

Supplementary Figure S1. Confocal pictures of the whole brains and CAs in *bombyxin>GFP* strains.

White and yellow dotted circles indicate cell bodies and CAs, respectively. Asterisks are non-specific signals derived from selection marker (3xP3-DsRed).

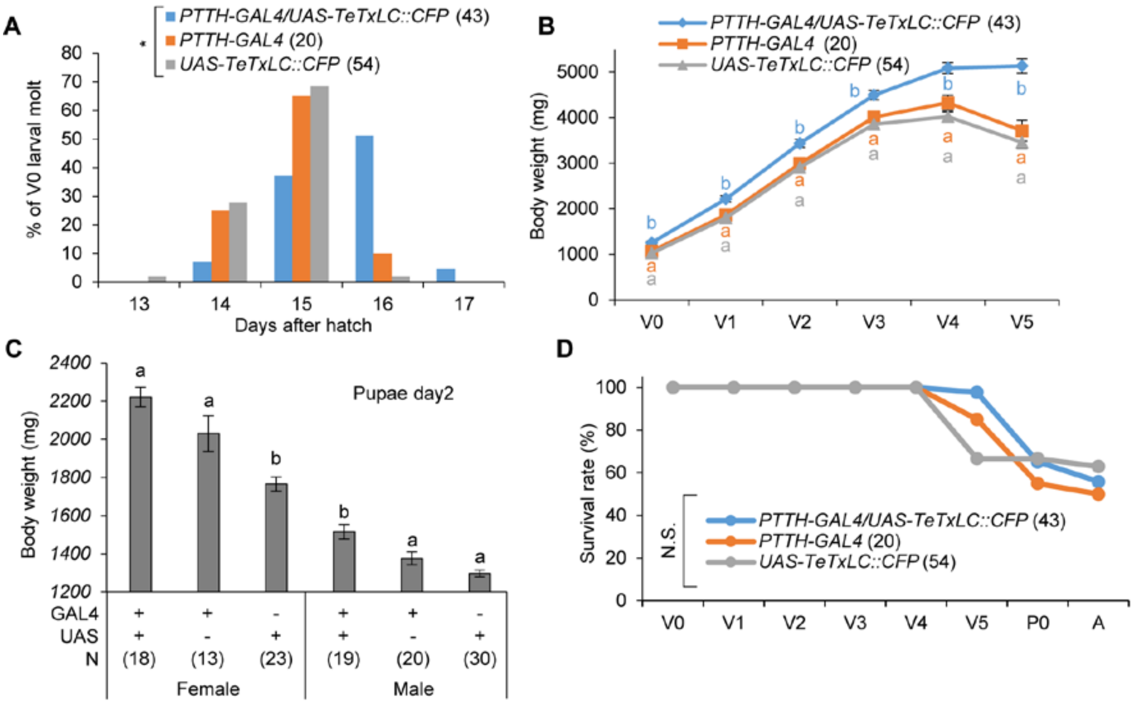

Supplementary Figure S2. Results of blockade of PTTH neural transmission in another batch.

(A) Timing of V0 larval molting. *: $P < 0.00001$, Chi-square test. (B) Developmental change in larval body weights. (C) Body weights in day 2 pupae. (D) Survival rate during larva-to-pupa development. Statistically different groups are shown in different characters ($P < 0.05$, *Tukey-Kramer*'s HSD test after ANOVA). N.S., not significant ($P > 0.05$). Number of samples are indicated in the parentheses.

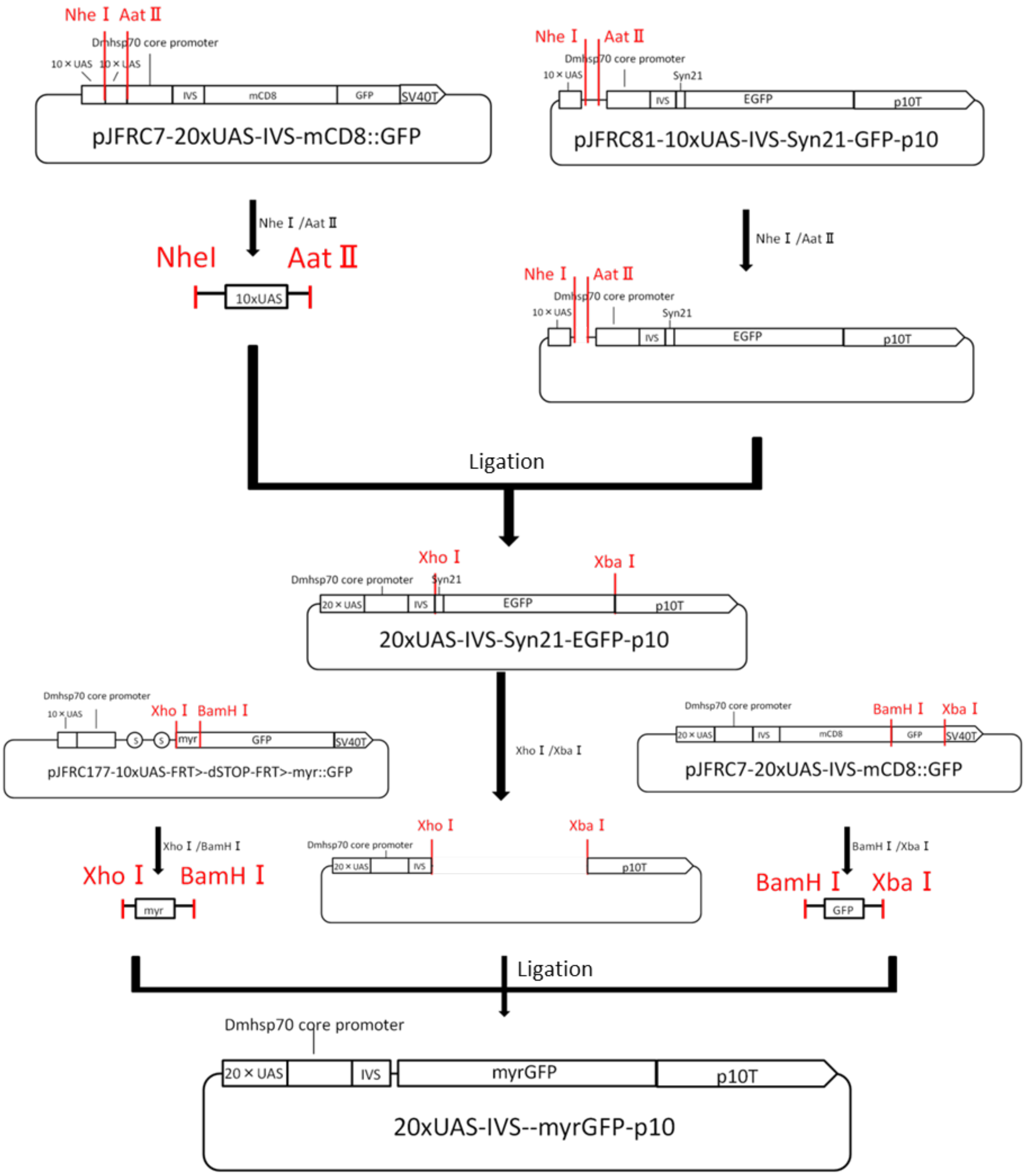

Supplementary Figure S3. Flow-chart of *20xUAS-IVS-myrGFP-p10T* construction.

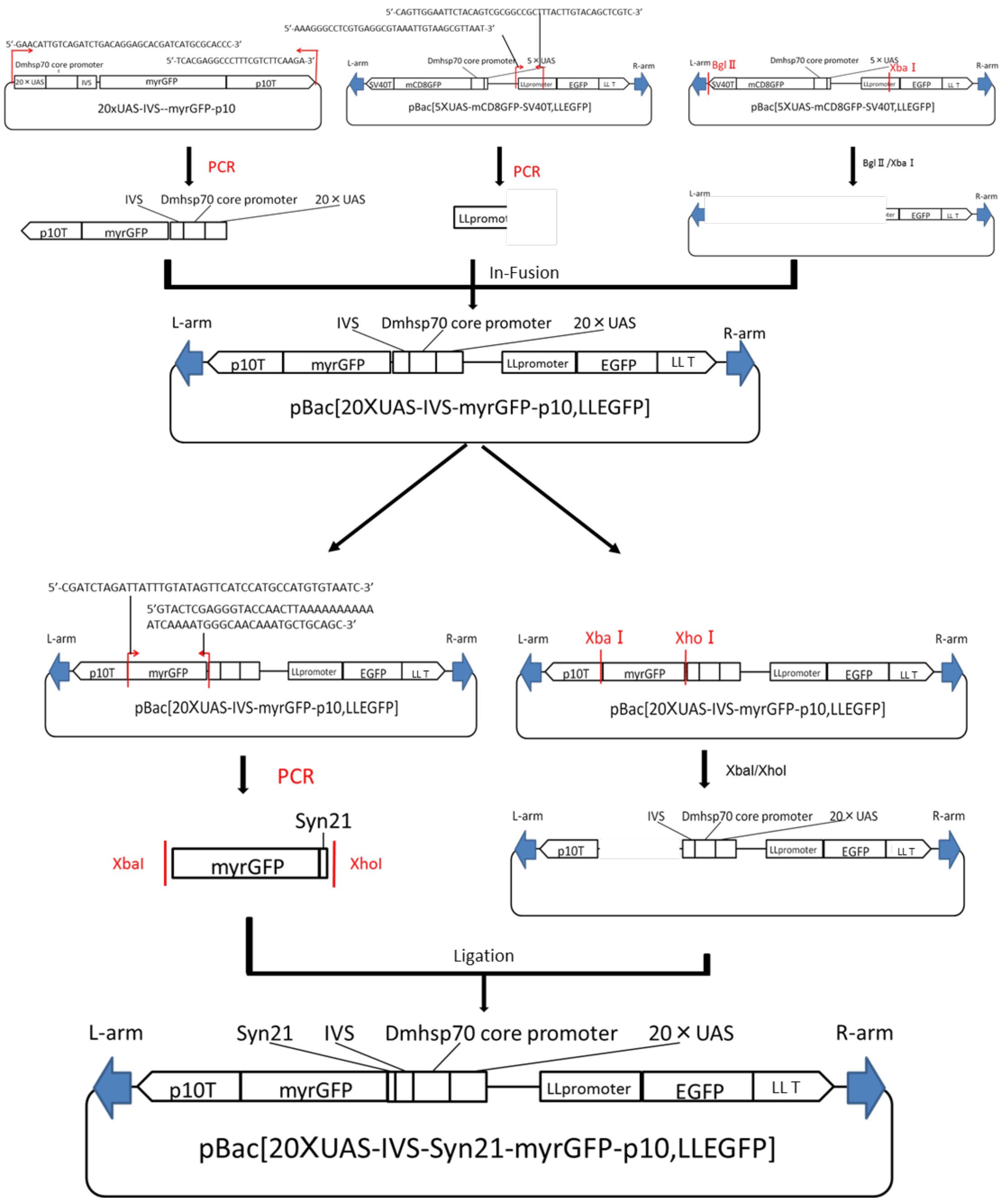

Supplementary Figure S4. Flow-chart of *PiggyBac*-based transformation vector *pBac [20xUAS-IVS-myrGFP-p10T, LL-EGFP]* construction.

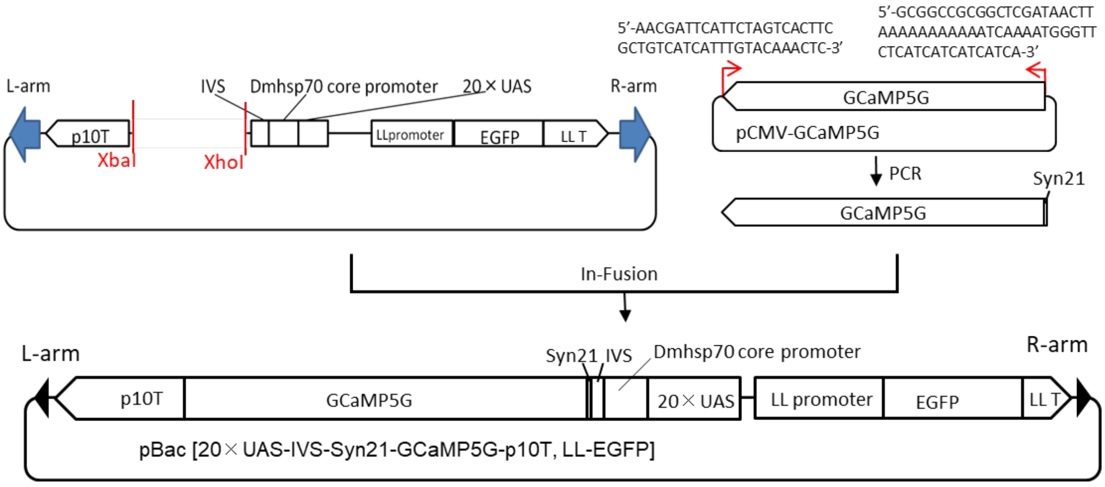

Supplementary Figure S5. Flow-chart of *PiggyBac*-based transformation vector *pBac [20xUAS-IVS-GCaMP5G-p10T, LL-EGFP]* construction.

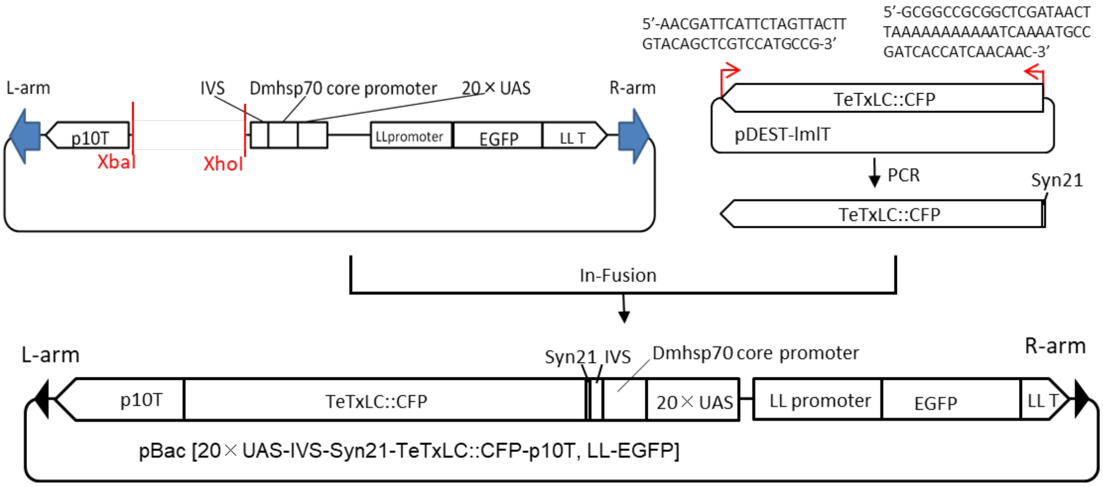

Supplementary Figure S6. Flow-chart of *PiggyBac*-based transformation vector *pBac [20xUAS-IVS-TeTxLC::CFP-p10T, LL-EGFP]* construction.
